# Importance of Mental Symptoms (Extract from *the Organon*)

**Published:** 1884-01

**Authors:** 


### IMPORTANCE OF MENTAL SYMPTOMS.

Although most homœopathic physicians tacitly acknowledge the great importance of mental symptoms, few, we believe, give them their just value. Hahnemann wrote (and it is well, once in awhile, to recall his advice): "The moral state of the patient is often the most decisive in the choice of the homœopathic remedy." To illustrate this he adds: "Aconite seldom or never effects a rapid and permanent cure when the patient is quiet and even; or Nux vomica, when the disposition is mild and phlegmatic; or Pulsatilla, when it is lively, serene, or obstinate; or Ignatia, when the mind is unchangeable and little susceptible to either fear or grief."

It is by attention to these little things—seemingly so meaningless, pathologically so useless—that the homœopathic physician achieves his grandest cures.

